# UCHealth’s virtual health center: How Colorado’s largest health system creates and integrates technology into patient care

**DOI:** 10.1038/s41746-024-01184-8

**Published:** 2024-07-11

**Authors:** Elizabeth Goldberg, Dave Kao, Bethany Kwan, Hemali Patel, Amy Hassell, Richard Zane

**Affiliations:** 1 University of Colorado Anschutz Medical Campus, Department of Emergency Medicine, Aurora, CO USA; 2 University of Colorado Anschutz Medical Campus, Department of Medicine – Cardiology, Aurora, CO USA; 3 University of Colorado Anschutz Medical Campus, Department of Medicine – Hospital Medicine, Aurora, CO USA; 4 UCHealth Nursing Administration, Aurora, CO USA

**Keywords:** Health policy, Medical research

## Abstract

In the face of formidable healthcare challenges, such as staffing shortages and rising costs, technology has emerged as a crucial ally in enhancing patient care. UCHealth, Colorado’s largest health system, has pioneered the integration of technology into patient care through its Virtual Health Center (VHC). In this Comment, we explore UCHealth’s journey in creating a centralized hub that harnesses innovative digital health solutions to address patient care needs across its 12 hospitals, spanning over 600,000 emergency department visits and nearly 150,000 inpatient and observation encounters annually. The VHC has proven to be a transformative force, providing high-quality care at scale, reducing staff burden, and establishing new career pathways in virtual health. The transformation process involved multiple steps: (a) identifying a need, (b) vetting within health system solutions, (c) searching for industry solutions, and scrutinizing these through meetings with our innovations center, (d) piloting the solution, and (e) sustaining the solution by integrating them within the electronic health record (EHR).

## Introduction

**Fig. 1:**
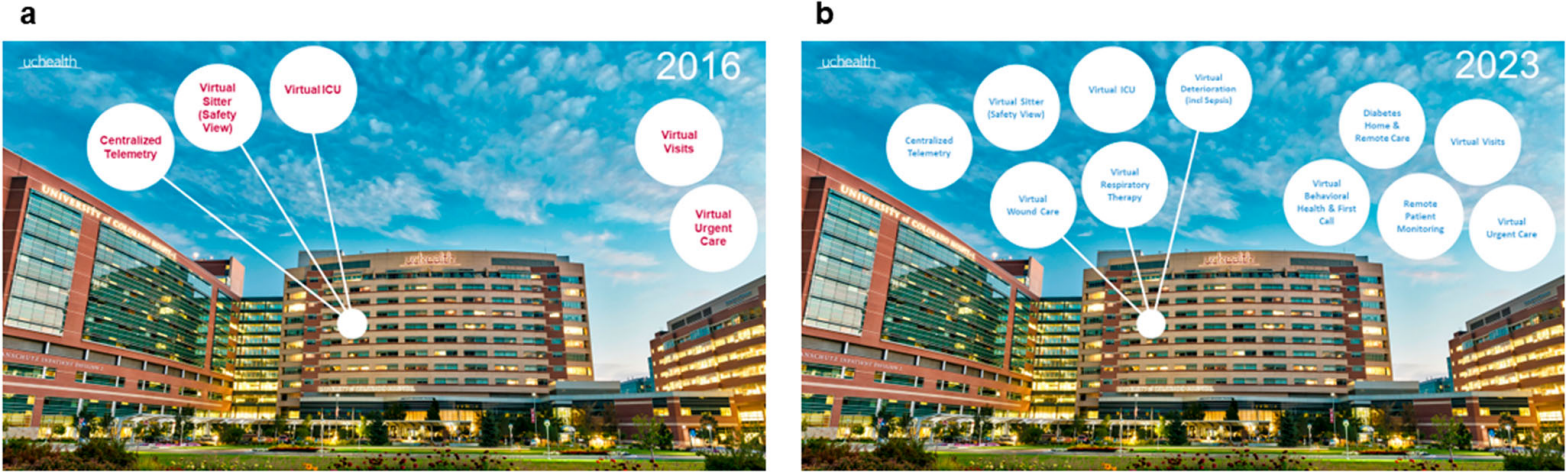
VHC. From 2016 **a** to 2023 **b**. In this two panel figure, we show the evolution of the Virtual Health Center. In 2016, we started the VHC by creating a business plan for three key hospitals. In 2016 we had <20 virtual visits for the entire month. By 2023, we scaled our virtual capabilities across all 12 hospitals. By 2023, we scaled virtual visits to over 70,000 a month.

UCHealth’s^1^ executive team, serving approximately 2.7 million patients across Colorado, recognized the need for a strategic approach to overcome challenges related to in-person care availability, especially in smaller facilities. In response, we established the VHC in 2016 (Fig. 1), initially focusing on critical care services in smaller and rural hospitals. Over time, it evolved to encompass myriad acute care functionalities, including remote telemetry, real-time predictive modeling, and virtual interventions^2^. We decided to invest in a centralized infrastructure that could work across multiple hospitals, vet existing technologies, and create new resources to meet our goals. This decision proved prescient during the COVID-19 pandemic, when rapidly increasing need for telemedicine services, unprecedented levels of staff illness, and challenges in expanding the clinical workforce made it essential to provide more support to patients with fewer resources. The VHC serves as a highly adaptable telemedicine platform for delivering multiple programs while achieving economies of scale (Table 1).

**Table 1:** Examples of how VHC programs achieve economies of scale

Program	Virtual telemetry	Virtual sitters (Virtual Safety View)	Virtual ICU
Details	• By centralizing cardiac monitoring, each of our telemetry technicians can monitor up to 42 patients at a time, compared to up to 10 patients for the traditional decentralized hospital-based model • Evolving prescriptive analytics will enable monitoring of up to 84 patients per technician within one year	• A centralized team of unlicensed technologists serve as “tele-sitters” and watch patients at risk of falling, with a 1:12 staffing ratio (compared to 1:1 staffing in a traditional model) • “Tele-sitters” redirect the patient verbally if they get up without support, and notify the nursing staff directly if in-person intervention is needed • Currently 12 tele-sitters are monitoring up to 144 patients at risk of falling at a time	• Centralized 24/7 Intensive Care Unit (ICU)-trained nurses provide ICU-level surveillance in medical/surgical (non-ICU) units • By integrating wearable and standard bedside monitor device data and feeding that data into algorithms within the EHR, the nurse in the VHC can watch patients and identify potential clinical decompensation • The VHC nurse then collaborates with bedside clinicians to initiate interventions • In 2017 one virtual ICU nurse watched 100 patients, now each nurse watches 1000 patients

### Key goals and achievements

The VHC’s mission includes delivering high-quality patient care, alleviating staff burden^3^, empowering clinicians, creating a virtual health career pathway, and seamlessly implementing new technologies. The center’s impact on access to care has been substantial, providing over 4100 interventions daily across more than 800 unique patients within and outside UCHealth’s 12 hospitals since inception.

### Infrastructure

**Fig. 2:**
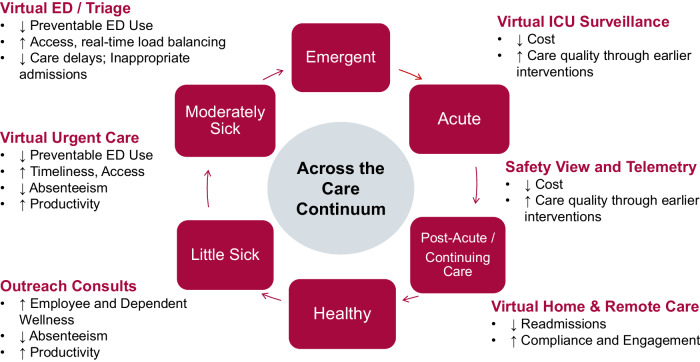
Virtual Health Center Care Continuum. The Virtual Health Center is comprised of several clinical teams focused on solving problems encountered within the health system.

The VHC is comprised of several clinical teams focused on solving problems encountered within the health system (see Fig. 2). Key aspects include (1) utilization of devices, algorithms, and the EHR (2024 EPIC Systems Corporation) to produce clinical intelligence such as risk models, decision support, and prescriptive recommendations; (2) operation of a physical VHC facility with sound dampening to ensure excellent two-way communication between centralized VHC staff and in-house clinicians. The facility is staffed 24/7 with technicians, nurses, and clinicians who use the technology, intelligence, and their clinical expertise to provide a “virtual safety net” for patients in beds across UCHealth; (3) health system leadership and a dedicated director with informatics and clinical experience, informatics, engineering, and analytics staff; (4) administrative teams to efficiently negotiate contracts with outside vendors who offer technological solutions to key clinical problems such as enabling one technician to monitor several patients at risk of falling across multiple facilities; and (5) EHR experts and other UCHealth engineers to create solutions where no viable commercial options exist. For example, if a product requires a clinician to navigate to an external website to use a risk calculator, we would instead use internal resources to build the calculator within our EHR. This allows the clinicians and staff to easily use the tool without requiring additional passwords, clicking and time.

### Clinical programs

The clinical programs are the core of the VHC and how it impacts patient care. Here we describe some of the programs and address what problems they solve for the health system.

### The virtual sitter program (safety view)

To address the challenge of monitoring patients at risk of falls efficiently, the VHC implemented an innovative remote video-capture technology. Creating the virtual sitter program began by vetting multiple companies to select a vendor. We learned from best practices from other hospitals about recommended staff to patient ratios and learned over time that we were able to change ratios so more patients could be monitored by one technician. We then codeveloped clinical criteria to determine what patient population was most likely to benefit. This Artificial Intelligence Risk of Injury Model includes history of falls, bleeding disorders, Epic’s fall risk score (FPM), frequent toileting, history of physical aggression, current alcohol withdrawal, cognitive impairment, frequent call light use, impulsive behavior as per nurse judgment, the AMPAC 6 clicks^4^, and current ICU patient.

Using this innovative technology, if a patient tries to get out of bed unassisted, the VHC technician can initiate an alarm and can verbally remind the patient to wait for assistance. As of July 2023, we can watch 144 patients for falls monitoring concurrently across all hospitals in the health system. Not all on-premises fall precautions can be replaced by video-audio monitoring. For instance, some patients may not be responsive to verbal requests to wait (e.g., due to poor hearing, dementia, delirium, or preferences). In these cases, the VHC staff may advise the unit nurses that they will need staff at the bedside. Additionally, we implemented this program with patient privacy and safety in mind; patients consent to the live stream and no recordings are generated.

### Virtual deterioration (Sepsis) program

The VHC’s early sepsis detection program leverages predictive models and EHR-embedded tools to identify and track impending sepsis. Previously published results indicate a reduction in sepsis-related mortality and improved time to antibiotics^5^. Goals for the virtual deterioration program included:

1. Standardize the identification and subsequent treatment of deteriorating patients with frontline staff and VHC tools
2. Provide timely identification of deterioration with frontline staff and VHC tools
3. Improve time from identified deterioration to time to intervention
4. Standardize vital sign monitoring after a rapid response when patient remains on the floor to detect further deterioration

Emergency and ICU trained physicians located in the VHC can remotely place orders such as intravenous fluids, antibiotics, and other essential, time-sensitive interventions necessary to treat sepsis in partnership with the primary bedside clinical team.

Several steps were necessary to successfully implement early sepsis detection in patients who were boarding (awaiting a hospital bed after being admitted from the emergency department): (1) VHC staff training to identify sepsis, (2) clinical intelligence applications such as predictive models to identify concerning clinical trends, and (3) change management among in-house clinicians to initiate interventions on patients determined by the VHC algorithms and staff to be at high risk.

**Fig. 3:**
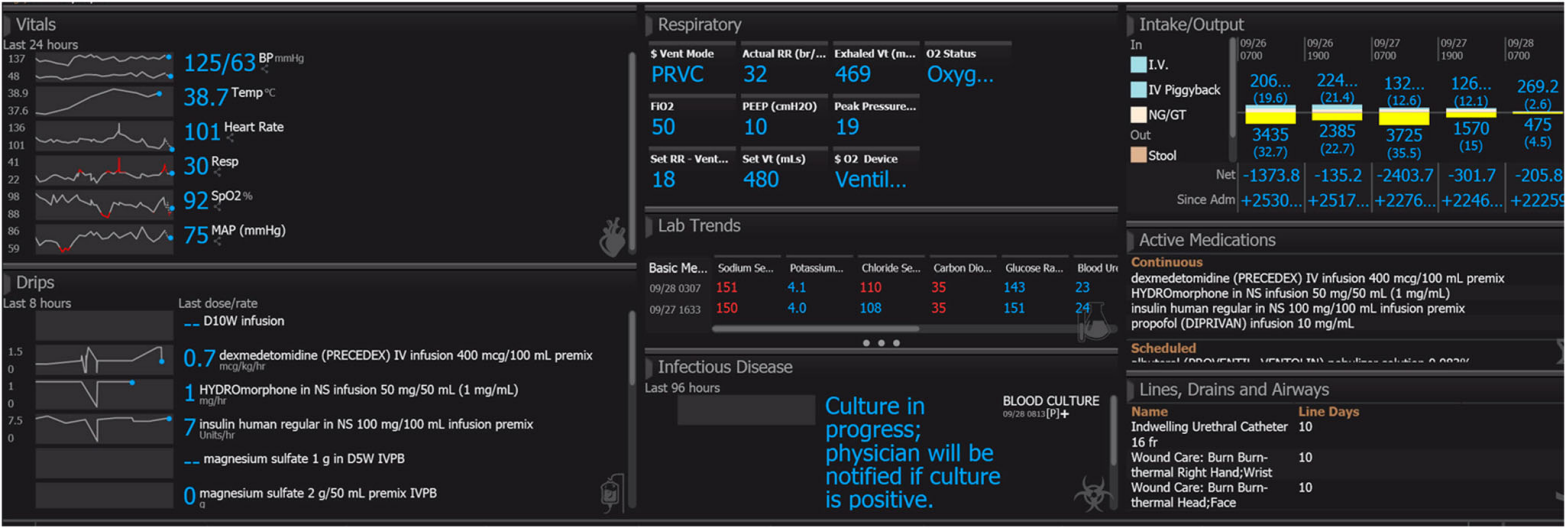
Early sepsis deterioration monitoring. Nurse dashboard to monitor metrics relevant to early detection of sepsis. In this figure, we show a nurse dashboard used to monitor metrics relevant to early detection of sepsis.

Elements of the clinical intelligence applications include: (a) a dashboard in the VHC that shows the current status of the four embedded predictive tools (Shock Index, Epic Deterioration Index, Epic Sepsis Prediction Model, and Respiratory Distress Index) and (b) modifications to the EHR to allow for efficient screening of patients across numerous hospitals within the health system. All predictive models were implemented using Epic’s Azure-hosted Nebula module using native Epic querying tools such as Reporting Workbench. There were no interfaces to outside resources. Therefore, components such as FHIR messaging and external standardized terminologies were not required for initial implementation. None of the predictive models are currently FDA approved. Our models use patient-specific information used in routine clinical practice to facilitate assessments, and these non-device Clinical Decision Support software functions are exempt from FDA approval^6^. The Epic Sepsis Model and Epic Deterioration index are proprietary algorithms licensed by Epic. The Shock and Respiratory Distress Indices are widely used, public-domain algorithms first published in 1967 and 1999, respectively^7^. Some of our predictive tools use continuous vital sign data from wearable devices applied to patients (e.g., we used the Sotera’s ViSi Mobile® Patient Monitoring System for early detection of sepsis). Using wearables changes the paradigm of monitoring from periodic vitals every four hours to continuous vitals, allowing our teams to act faster when vital signs change. Nurses can visualize data including vital signs, drips, respiratory metrics, and other relevant parameters on a dashboard (see Fig. 3). VHC nurses can review these data for 50–75 patients per shift.

### Clinical programs relevant to COVID-19

During the COVID-19 pandemic, the VHC played a crucial role in triaging ill employees, scaling virtual urgent care and visits, and implementing a home monitoring program for stable patients with COVID-19. The VHC provided virtual employee health assistance by triaging employees who required testing, isolation, and return to work guidance. We scaled virtual visits from <1000 visits a month in the month prior to the pandemic onset across primary care, specialty care, and urgent care to over 70,000 virtual visits per month.

We also created a program for home monitoring of discharged stable inpatients who had COVID-19. The VHC monitored patients in the program for up to eight days during which they were weaned off oxygen. This program allowed us to achieve early discharge for patients with COVID-19 and preserve inpatient capacity^8^. Using a two-to-one-matched case-control design, we compared patients who were discharged with remote patient monitoring from March 2020 to February 2021. Patients discharged were matched on age, sex, Charlson comorbidity index, and limited English proficiency. Overall, 203 patients were enrolled representing 1293 patient-days of monitoring. Of these, mean age was 53 years (SD 15), 42% had limited English proficiency, and 70% percent were discharged with home oxygen supplies. Those who were discharged with remote patient monitoring had a decrease in length of stay without increases in 30-day emergency department revisits or hospital readmissions. Although the association between discharge with remote patient monitoring and decreased length of stay was not significant in adjusted analysis, we observed a trend for reduced length of stay.

We did not experience major technology problems with the home monitoring program. The biggest issue we had was that patients lacked data plans and relied on public Wi-Fi. For this program, we provided smartphones to patients at enrollment to address health equity. We also at times experienced problems pairing the wearable.

### Centralizing and optimizing: the virtual sandbox

Finding technology partners and creating in-house technological solutions can be challenging, but the most critical element of successful implementation has been driving adoption and focusing on change management (see Table 2). Centralizing capabilities and unifying approaches require buy-in from clinical partners, socialization among end-users, and demonstration of improvements in efficiency and outcomes. Several questions have arisen from the community during the process of implementing VHC solutions including patient privacy concerns, medicolegal concerns, overlap in clinical responsibility between services, role of clinicians vs. technology-based recommendations, and data sharing.

**Table 2:** Change management: Our approach to implementing technology-enabled solutions in healthcare

Strategy	Approach
Clear description and acceptance of problem statement	Using unambiguous performance data at the unit- and provider-level demonstrated that the current state of performance was unacceptable.
Demonstrating accuracy in monitoring and surveillance	Every single case was communicated to the local teams, reviewed for accuracy and timing, and then followed. As an example, when local teams disagreed with the VHC’s assessment of sepsis, results were shared which clearly demonstrated accuracy of the VHC’s diagnosis of sepsis.
Gradual change in decision making: “Opt in vs. Opt out”	Initially, the VHC would inform local teams of a deteriorating patient and follow outcomes. When it was clear that the VHC was accurate and local teams could not act as quickly, the VHC would inform local teams of patients’ condition and the remote interventions initiated. The local teams could evaluate the patient and change the orders or allow the orders to stand.
Celebrating wins and earning buy-in from clinical partners	Rigorous evaluation and sharing of impact (pre and post), celebrating early adopters and wins; creating an innovation culture
Socialization among end-users	Demonstrations by nurse managers and physician leaders of new dashboards, analytic approaches, and clinical workflows
Top of scope ethos	By having the VHC perform functions remotely it allows nurses, advanced practice providers, and physicians locally to work at the top of their scope
Demonstration of efficiency and outcomes	Creating a centralized VHC team with expertise in data science, analytics, EHR builds, interoperability; demonstrating success and ease of use; application/interface design to create-enhanced efficiencies; time savings when using new applications compared to existing
Post-implementation revision and improvement	Provide opportunities for feedback from end-users and follow-up to improve applications
Patient privacy and ethical concerns	Engaging compliance and bioethics leaders to ensure a process for consent, opting out, and communication with key partners (e.g., patients, families, clinicians)

Overcoming these concerns requires excellent engagement of partners, frequent communication, and transparent sharing of evaluation data. By employing these principles, several teams have been able to demonstrate cost reductions, gain efficiencies, and improve outcomes, which created momentum and more growth of the VHC. A key advantage of the VHC is that it acts as a sandbox for experimentation with a smaller staffing level, which allows for easier outreach and education, and the means to test new initiatives in a centralized location among experienced staff prior to scaling it to all sites.

Here, we focus on the successes of the program, but we have also encountered challenges implementing the VHC. At times we had to retire technology that did not work. We invested staff time and effort into vetting, implementing, and evaluating technology tools. We recommend organizations stand up a team of experts knowledgeable in health technology and industry partnerships with direct communication to the health systems’ executives, to address this. We remain challenged by staff recruitment and retention in the VHC. Just as in in-person settings, there is a shortage of qualified staff to work in tele-healthcare. We have dedicated VHC directors who lead recruitment and reduce turnover. Change management also remains a challenge—while everyone has a central value of patient safety, change is inherently difficult for anyone and therefore innovation programs require a particular focus on early adopters to drive short-term wins and lead to more global changes over time. We have a robust analytics team, but are still working on optimizing turnaround time for analytics requests and have prioritized clinical needs over research and dissemination.

### Dissemination: from concept to health system

UCHealth aims to share the success of the VHC with the broader healthcare community, disseminating best practices and insights. Recommendations from our VHC team of experts based on experience implementing these programs for the past eight years include: (1) identify an innovations leader who has direct communication with the healthcare system C suite. The director should have a clinical background and experience with operations and informatics approaches, (2) employ ICU nurses as they are ideally suited to receive and interpret a wide range of clinical acute care data and share these with on-site teams, (3) employ emergency department and hospital medicine physicians with experiences in triaging to make hospital transfer decisions, and (4) build a team with strong clinical analytics and engineering expertise. Over time we expect to integrate more advanced wearable technology and devices, and produce even better algorithms and intelligence in the inpatient and outpatient environments. We are planning a Virtual Primary Care and Transitions of Care program. We are also piloting Virtual Admission and Quality workflows.

We also acknowledge that the use of precision medicine and predictive analytics is not unique to our own health system. For instance, academicians at Yale created a machine learning tool to identify emergency department patients near the end of life^9,10^, University of Wisconsin models predict patients at future risk of falls to guide fall prevention referrals^11^, and researchers at Cincinnati Children’s Hospital have created a point-of-care tool to identify patients with cystic fibrosis who are likely to have a rapid clinical decline^12^. We are not aware of other health systems reporting on the creation of centralized virtual health centers.

## Conclusion

Over the past eight years, UCHealth’s VHC has emerged as a transformative approach to healthcare in our system, supporting clinicians, achieving efficiencies, and positively impacting patient outcomes. The VHC’s innovative approach serves as a beacon for healthcare systems seeking to integrate technology seamlessly into patient care, providing a scalable model for the future.

## Data Availability

All data generated or analyzed during this study are included in this published article and it’s referenced articles.
